# Effects of pressure angle and tip relief on the life of speed increasing gearbox: a case study

**DOI:** 10.1186/2193-1801-3-746

**Published:** 2014-12-16

**Authors:** Sankar Shanmugasundaram, Manivarma Kumaresan, Nataraj Muthusamy

**Affiliations:** Department of Mechanical Engineering, Nehru College of Engineering and Research Centre, Thrissur, India; Department of Mechanical Engineering, Sri Krishna College of Engineering and Technology, Coimbatore, India; Department of Mechanical Engineering, Government College of Technology, Coimbatore, India

**Keywords:** Failure analysis, Helical gear, Wind turbine gearbox, Profile modification, Bending stress, Tooth deflection, KISS soft, ANSYS

## Abstract

This paper examines failure of helical gear in speed increasing gearbox used in the wind turbine generator (WTG). In addition, an attempt has been made to get suitable gear micro-geometry such as pressure angle and tip relief to minimize the gear failure in the wind turbines. As the gear trains in the wind turbine gearbox is prearranged with higher speed ratio and the gearboxes experience shock load due to atmospheric turbulence, gust wind speed, non-synchronization of pitching, frequent grid drops and failure of braking, the gear failure occurs either in the intermediate or high speed stage pinion. KISS soft gear calculation software was used to determine the gear specifications and analysis is carried out in ANSYS software version.11.0 for the existing and the proposed gear to evaluate the performance of bending stress tooth deflection and stiffness. The main objective of this research study is to propose suitable gear micro-geometry that is tip relief and pressure angle blend for increasing tooth strength of the helical gear used in the wind turbine for trouble free operation.

**Electronic supplementary material:**

The online version of this article (doi:10.1186/2193-1801-3-746) contains supplementary material, which is available to authorized users.

## Introduction

The function of gear drive is to transmit high power with compact design as to run with free of noise and vibration with least manufacturing and maintenance cost. Sankar and Nataraj have introduced circular root fillet instead of trochoidal root fillet in spur gear to increase the tooth strength (Sankar and Nataraj 2011). Many works have been done to improve the gear tooth strength out of which most of them attempted with positive profile shifting (Fredette and Brown 1997; Ciavarella and Demelio 1999). Sankar and Nataraj have launched a novel method called composite profile along with tip relief in helical gear to prevent gear failure in the wind turbine generator gearbox (Sankar and Nataraj 2010). Andrzej and Jerzy have done a comparative study to evaluate root strength using ISO and AGMA standards and the results are verified using the finite element technique with model development and simulations (Andrzej and Jerzy 2006). Simon formulated a design method to find out the optimal tooth tip relief and crowning for spur and helical gears (Simon 1989). Sankar et al. (2011) have formulated mathematical model to analyze the failure of shear pin in the wind turbne generator using finite element technique. Hebbal et al. (2009) have formulated a finite element model with a segment of three teeth for analysis and stress relieving features of various sizes on helical gear teeth at various locations.

Senthilvelan and Gnanamoorthy (2004) have evaluated the gear performance with the help of finite element analysis using a power absorption type gear test rig.

Mao established the gear micro geometry modifications mathematically for power train gear transmission using python script interfaced with finite element models (Mao 2006).

Jiande Wang and Ian Howard (2008) demonstrated the influence of high-contact-ratio spur gears in mesh with tooth profile modification by using modern numerical methods via comprehensive analysis. Tae et al. (2001) discussed tooth modification for minimizing the vibration exiting force and noise in helical gears. Beghini et al. (2004) proposed a method to reduce the transmission error of spur gear at the normal torque through profile modification parameters. Satoshi et al. (1988) studied the effect of standard pressure angle on the bending strength of helical gears by using the approximate equation to a considerable extent. Satoshi et al. (1986) analyzed the tooth deflection and bending moment at the root fillet in helical gear for various pressure angles by finite difference method. Shan Chang et al. (2005) used tip relief and root relief to reduce the high contact stresses occur at the root corners in the entering and exiting regions. Alexander et al. (2003) presented a novel method for bending stress balance using one-hundred-year-old Lewis equation suggesting an approach to the tooth parameter’s tolerance and tooth profile definition.

In general, tooth profile modification methods are used to reduce the meshing vibration and noise of gear train. Kinds of such methods are (i) Tooth profile modification towards involute curve (ii) Lead crowning and End relief towards face width and so on. Many research papers have been published towards reducing noise and vibration of spur gears by make use of tooth profile modification towards involute curve but an attempt has not been made to propose simultaneous optimum profile modification towards involute curve for various pressure angle of helical gears employed in the wind turbine generator gearbox, to the best knowledge of the investigator. Therefore, it is imperative to investigate the problem of failure of helical gear in the wind turbine.

## Case description

**Figure 1:**
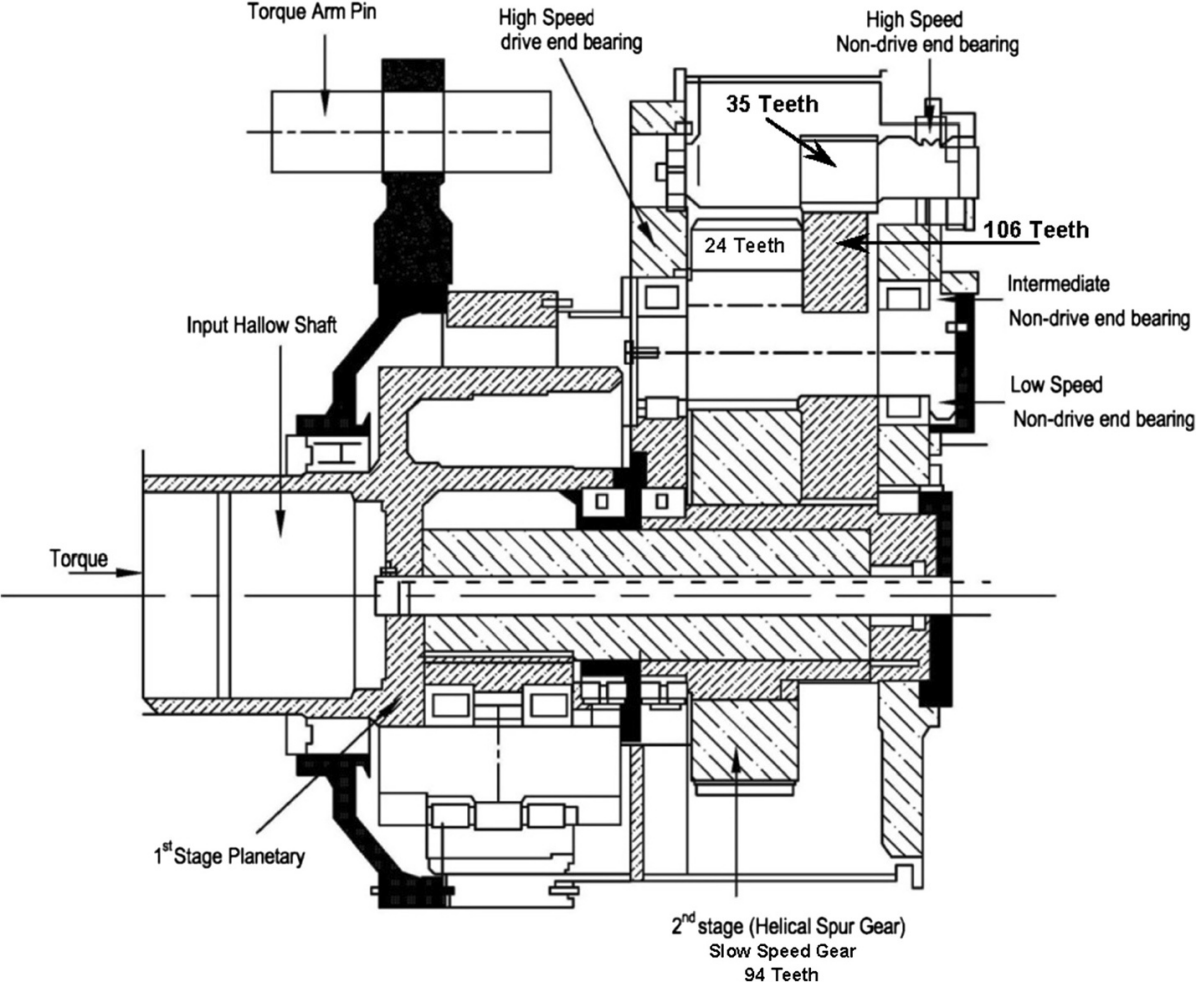
**Sectional view of the wind turbine generator gearbox.**

The particular model wind turbine generator is built with gearbox comprising of one planetary stage and two helical stages. The first helical stage called slow speed line has 94 teeth/24 teeth gear combination and the second helical stage called high speed line has 106 teeth/35 teeth gear combination to get the final rated speed of an electric generator. This speed increasing gearbox raises abnormal noise, have scuffing wear and pitting wear, during peak generation of the wind turbine in high wind season, which ultimately leads to either tooth damage or failure of pinion itself. Besides, if any pinion in the intermediate stage undergone failure, while the wind turbine generator is running, it’s horrible to swap the pinion alone at tower top (nacelle) at the wind turbine site due to complication in the gearbox design. At this point of time, the only available solution is de-erection of the nacelle for swapping the gearbox. Moreover, for de-erection of nacelle a huge capacity crane (400 or 800 Ton capacity) is required at wind turbine site for swapping the gearbox. The sectional view of the wind turbine generator gearbox is depicted in Figure 1.

**Figure 2:**
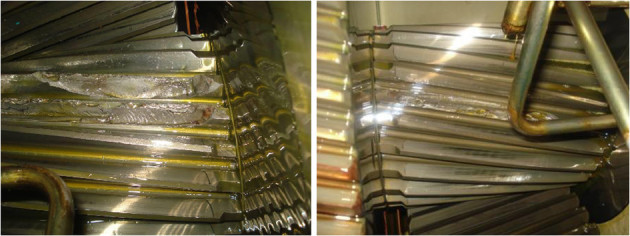
**Failed pinions.**

In the past 2-3 years, the wind turbine site come across numerous failures of 24 teeth pinion which is coming in 94 teeth/24 teeth gear combination. Figure 2 shows two different cases of 24 teeth intermediate pinion failure happen at the wind turbine site recently. The gear pair is made of 8 mm module having 20° pressure angle with tiny say 0.002 mm tip relief. The technical team inspected the damaged pinion (Figure 2) and presumed that the failures may be due to either overload by wind force or misalignment of shaft between the gearbox and the generator. Gear manufacturer and researchers are exploring the possibilities either on development of advanced materials such as 3Ni-4.5Mo alloy and 3Ni-2Cu alloy (Popgoshey and Valori 2009) new methods of heat treatment for gears such as Low Pressure Carburizing (LPC) with high pressure gas quench and Press Quenching of Gears (Nicholas Bugliarello et al. 2010) or on the design of stronger tooth profiles (Sankar et al. 2011) and on the new gear manufacturing process. This research study is intended to know the root cause of failure of pinion and to minimize to minimize the pinion failures in gearboxes used in the wind turbines through design modification such as pressure angle and the tip relief.

## Geometrical modeling

### Geometry of the tip relief

**Figure 3:**
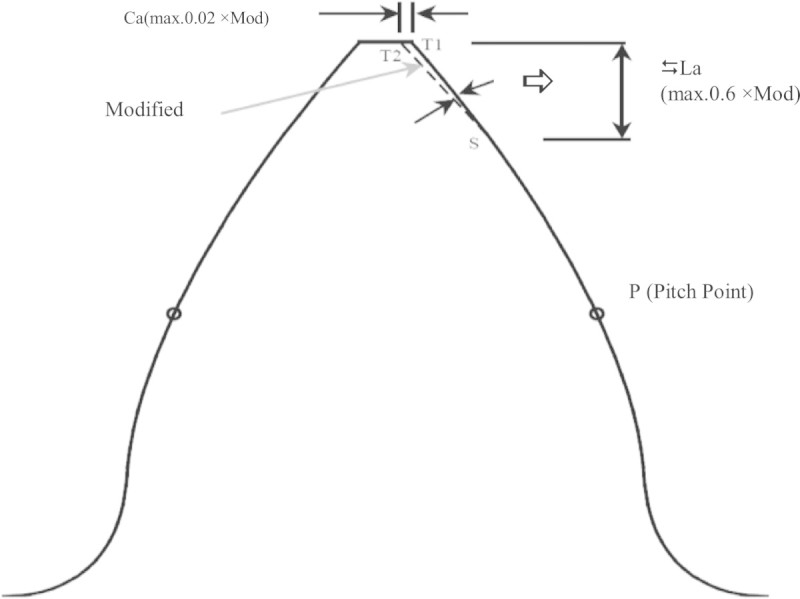
**Gear profile with tip relief.**

Tip relief is discretionary modification of the tooth profile near the tip of the tooth to eliminate tip interference. It is considered desirable for the involute to be a few thousandths minus at the tip and never plus. Tip relief is given to gears during gear grinding operation through dressing or truing of grinding wheel with the help of special diamond disc in case of multi rib grinding wheel and single point diamond dresser with special template for single rib grinding wheel. The conventional amount of tip relief is given in the existing standards like British Standard (BS 1970) and (ISO/DIS 1983), where the maximum amount of tip and flank modifications are defined as shown in Figure 3, including parameters such as maximum amount of tip relief (C_a max_) = 0.02 times of module and maximum length of tip relief (∆L_a max_) = 0.6 times of module to prevent the possibility of excess relief. In Figure 3, where

$$\begin{array}{lll} C_{a} & - & \begin{array}{l} \mathrm{permissible}\;\mathrm{tip}\;\mathrm{relief}\;\mathrm{amount}\;\mathrm{near}\;\mathrm{tip}\;\mathrm{of}\;\mathrm{gear}\\ \left(C_{a\;\max}=0.02\times \mathrm{module}\right) \end{array}\\ \Delta L_{a} & - & \begin{array}{l} \mathrm{allowable}\;\mathrm{relief}\;\mathrm{length}\\ \left(\Delta L_{a\;\max}=0.6\times \mathrm{module}\right) \end{array} \end{array}$$

**Figure 4:**
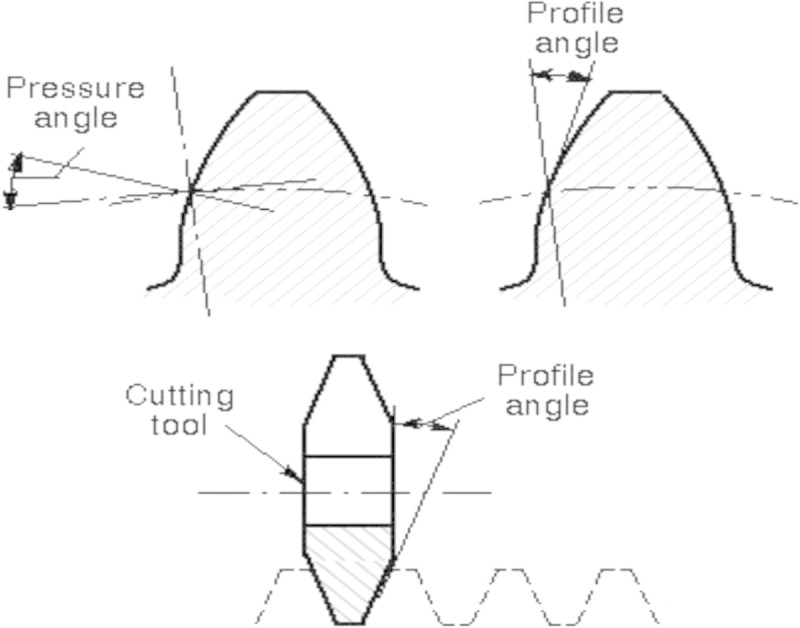
**Gear pressure angle.**

In this study the standard tip relief limitations have been chosen as reference values to normalize the amount of profile modification. There are two different tip relief methods exist for profile modification which are (i) Linear and (ii) Parabolic variations. The modified profile form used in this research involves the original involute and the relief was achieved by rotating the original curve through relief angle ‘α_r_’ about the relief starting point ‘S’ as shown in Figure 3. Pressure angle is the angle between the tooth profile and a perpendicular to the pitch circle usually at the point where the pitch circle meets the tooth profile as shown in Figure 4. The pressure angle affects the force that tends to separate mating gears.

A high pressure angle means that higher ratio of teeth are not in contact. However, this allows (i) the teeth to have higher load carrying capacity (ii) allows less number of teeth without undercutting (iii) tooth flank becomes more curved and hence relative sliding velocity is reduced (iv) the tooth pressure and axial pressure is increased. (v) Increase of pressure angle results in a stronger teeth, because the tooth acting as a beam is wider at the root (Sankar and Nataraj 2010). This analysis is carried out for three different pressure angles say 15°, 20° and 22.5° for various tip relief length and amount.

## Part modeling

Table 1 gives the design specifications of the existing 24 teeth helical pinion and Table 2 gives the design specifications of the modified 24 teeth helical pinion. These design specifications have been arrived from KISS soft software according to DIN 3990 method ‘B’ standards. According to KISS soft gear calculation software, the addendum and dedendum values can be interchanged for the mating gear pair having correction factor. As the addendum of the pinion may be more than one module and the dedendum has been reduced to less than one module.

**Table 1:** **Design specifications of the existing pinion**

Number of teeth (z)	Pinion	24	Center distance (a)		485.00 mm
	Gear	94	Tip diameter (d_a_)		219.52 mm
Normal module(M_n_)	8 mm		Addendum modification co-efficient (x)	Pinion	0.56 mm
Pressure angle (α)	20°			Gear	0.18 mm
Helix angle (β)	10°		Root diameter (d_f_)	184.00 mm	
Face width (b)	245 mm		Addendum (h_a_)	12.278 mm	
Hand of helix	Right		Dedendum (h_f_)	5.481 mm	
Reference diameter (d)	194.96 mm		Effective chordal tooth thickness	15.744/15.694	
Base diameter (d_b_)	182.872 mm		Total contact ratio	2.948	
Tooth quality (Q-DIN3961)	6		Tip relief ( C_a_)	0.002 mm

**Table 2:** **Design specifications of the modified gear pair**

No of teeth	Pressure angle	Tip relief (mm)
Pinion 24	15° and 22.5°	$\begin{array}{c} \mathrm{Case}\left(i\right)C_{a}=0.08\\ \Delta L_{a}=2.40 \end{array}$
Gear 94		$\begin{array}{c} \mathrm{Case}\left(ii\right)C_{a}=0.16\\ \Delta L_{a}=4.8 \end{array}$

## Profile modification

**Figure 5:**
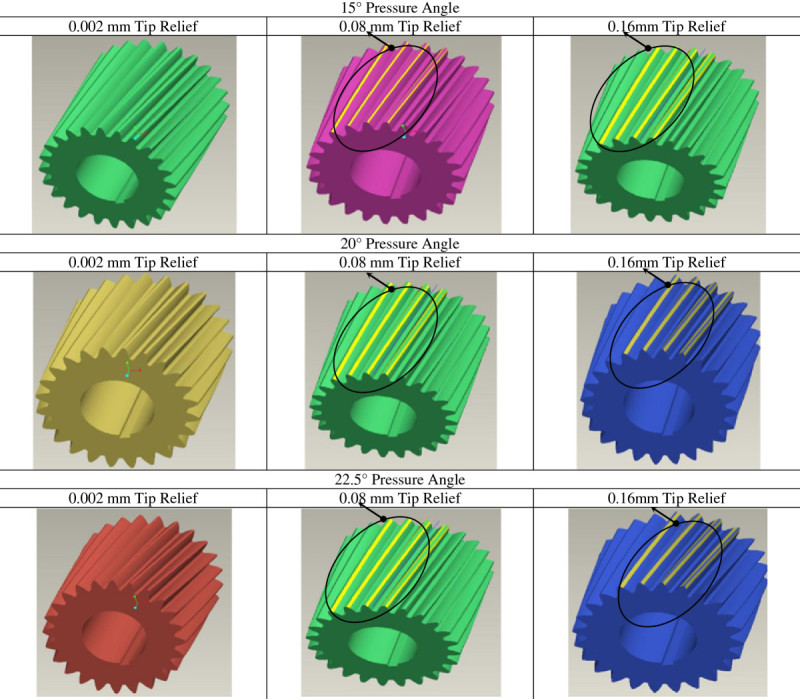
**Pro-E models of the modified 24 teeth pinion.**

The tip relief is introduced in the pinion profile for the corresponding change in pressure angle as given in Table 2. The models with appropriate tip relief with respective pressure angles generated through Pro-E wildfire version 3.0 software are presented in Figure 5.

## Force analysis

**Figure 6:**
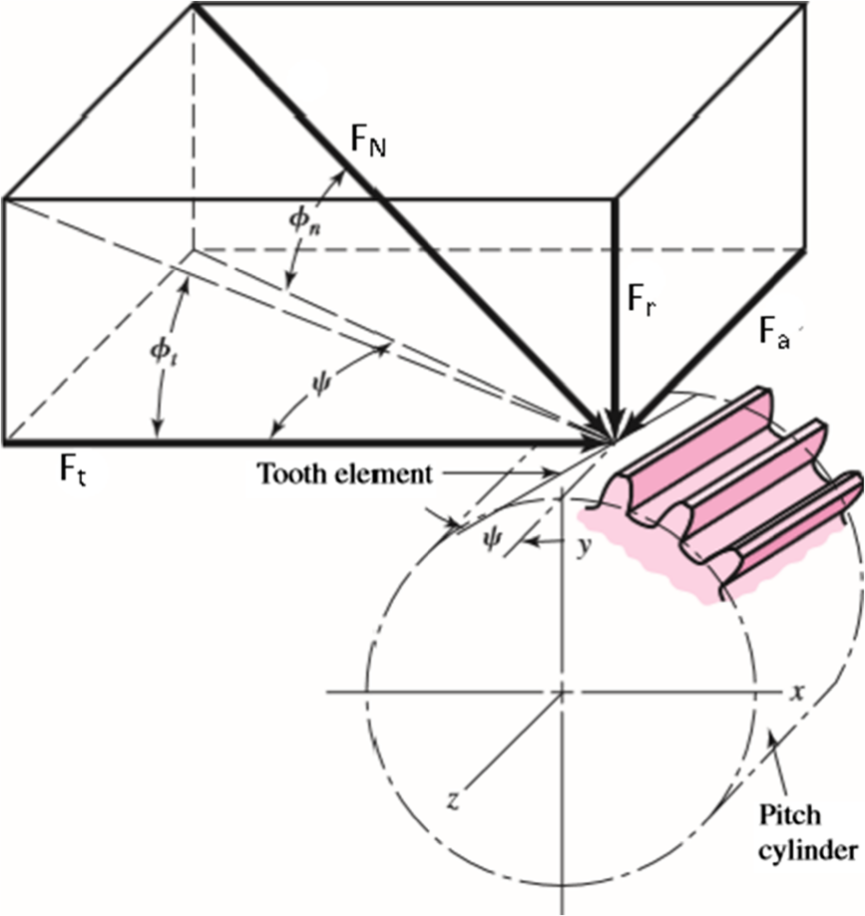
**Tooth forces in helical gear.**

Force analysis for helical gears can be made in similar manner as in the case of spur gears (Sankar and Nataraj 2011). Because of the helix angle, an additional force component is produced. This appears as an axial force with the resulting axial thrust on the bearings. The pictorial view of helical gear tooth forces is shown in Figure 6. In helical gears tooth force F_N_ acts normal to the tooth surface at an angle equal to the pressure angle. This tooth force is resolved into three components which act at right angles to one another. The interrelations of these components are established from Figure 6. The three dimensional force patterns are obtained with their magnitudes which are shown below (Equation 1 to 5):

1 $$\mathrm{Tangential}\;\mathrm{force}\left(F_{t}\right)=2000T/d$$

2 $$\mathrm{Radial}\;\mathrm{force}\left(F_{r}\right)=F_{n}\sin\alpha$$

3 $$\mathrm{Axial}\;\mathrm{force}\left(F_{a}\right)=F_{t}\times \tan\beta$$

4 $$\mathrm{Normal}\;\mathrm{force}\left(F_{n}\right)=F_{t}/Cos\alpha \times Cos\beta$$

5 $$\mathrm{Power}\left(P\right)=T\times N/9549\mathrm{in}\;\mathrm{kW}$$

Where,

α: Pressure angle

β: Helix angle

N: Speed in rpm

d: Pitch circle diameter in mm

T: Driving torque in Nm

While the helical gear pair is transmitting the load, the leading end of the tooth comes in contact first and the trailing end last. Thus the tooth picks up load gradually and the contact progresses gradually along the whole range of the tooth width that is in helical gear pair, sharing of load will take place based on the contact ratio, covering the tooth face and flank.

In actual practice, trochoidal root filet is formed in gears during manufacturing process depending on the tip radius of the hob. It was proved that the bending stress decreases gradually in gears as the number of teeth increases and the total contact ratio increases (Spitas et al. 2005). According to Gitin Maitra, if a gear is undercut for one reason or another, it may become sometimes necessary to know the magnitude of the undercutting radius (Gitin Maitra 1998). Under such circumferences, he proposed a formula (Equation 6) to find out the minimum number of teeth to avoid undercutting which is as follows:

6 $$Z\min=2/{\mathrm{Sin}}^{2}\alpha$$

Referring to the above equation, the minimum number of teeth to avoid undercut problem for 15° pressure angle pinion is 30. Similarly, it is 17 and 14 for 20° pressure angle pinion and 22.5° pressure angle pinion respectively. Further, the above expression is valid for standard gear tooth with the addendum of the rack being equal to the module ‘Mn′. However, the undercut–free minimum number of teeth is given by Equation 7.

7 $$Z_{\text{min}}=2h_{ca}/Mn\;{\mathrm{Sin}}^{2}\alpha$$

Where, h_ca_ is the addendum of the rack cutter without tip filet rounding. It is obvious from KISS soft gear calculation (Table 3) that the addendum and dedendum of the in-use 20° pressure angle pinions is 12.279 mm and 5.481 mm respectively. Similarly, the addendum and dedendum of its mating gear are 9.20 mm and 8.56 mm respectively. So, if addendum of the cutter is 5.481 mm without tip fillet rounding then based on Equation 7, the minimum number of teeth to avoid undercut–free operation on 20° pressure angle pinion is 12. So, it is very clear that the undercut risk is carefully considered in this 20° pressure angle design and hence the pinion number of teeth is chosen as 24. In the same way, the addendum and dedendum of the 15° pressure angle pinions is 12.278 mm and 5.288 mm respectively. Also, it is 9.006 and 8.56 mm for its mating gear. So, if addendum of the cutter is 5.288 mm without tip fillet rounding then based on Equation 7, the minimum number of teeth to avoid undercut–free operation for 15° pressure angle pinions is 20. But, in these study only 24 teeth was considered for the entire model. So it is very clear from the study that the 15° pressure angle pinion does not have an undercut problem. Besides, according to shigley, the minimum number of teeth to avoid interference for 20° pressure angle full depth profile is 17. Similarly, it is greater than 23 for 15° pressure angle pinion (Shigley 2008). So, the modified design would not face any interference problem too.

**Table 3:** **Tooth details from KISS soft gear calculation**

No of teeth (z)	Pr angle (α)	Add (h_a_)	Ded (h_f_)	Centre distance(a)
24	15	12.278	5.288	485.00
	20	12.279	5.481	
	22.5	12.278	5.535	
94	15	9.006	8.560	
	20	9.200	8.560	
	22.5	9.253	8.560	

## Force calculation

The force exerted by the helical pinion on its mating gear acts normal to the contacting surface if the friction is neglected. However, a normal force in case of helical gear has three components that is apart from the tangential force (F_t_) and radial force (F_r_) that are present in spur gear, a third component parallel to the axis of the shaft called axial force (F_a_) or thrust force exists. These components of force are computed for a power value of 1252 kW at pinion speed of 509.2 rpm. These values are given in Table 4.

**Table 4:** **Force components of the load**

Pressure angle	Torque (Nm)	Force components (N)			
		(F_t_)	(F_n_)	(F_a_)	(F_r_)
15°	23443	240492	252817	42405	65433
20°	23443	240492	259874	42405	88882
22.5°	23443	240492	264322	42405	101152

As far as the transmission power is concerned, the tangential force (F_t_) is really the useful component, because the radial force (F_r_) and axial force (F_a_) serves no useful purpose. Hence, only the tangential force was applied in the entire model say 15°, 20° and 22.5° for evaluating the performance in FEA using ANSYS.

## Finite element analysis

**Figure 7:**
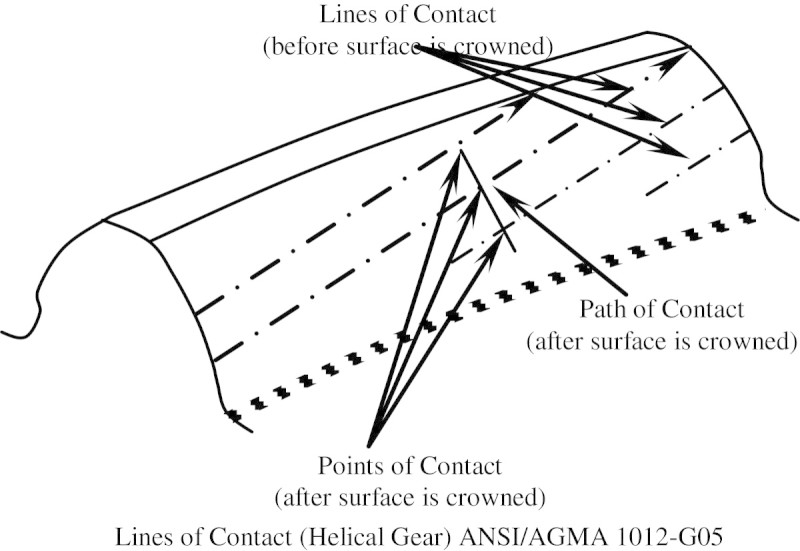
**Line of contact in helical gear.**

In this study finite element model with a single tooth is considered for analysis. Gear material strength is major consideration for the operational loading and environment. Generally cast iron is used in normal loading and higher wear resisting conditions. In modern practice, the heat treated alloy steels are used to overcome the wear resistance. In this work, carburized and case hardened alloy steel (17CrNiMo6) is considered and ANSYS version 11.0 software is used for analysis. According to ANSI/AGMA 1012-G05 standard as in Figure 7, the strength analysis is carried out for the traditional and the modified 24 teeth pinion.

**Figure 8:**
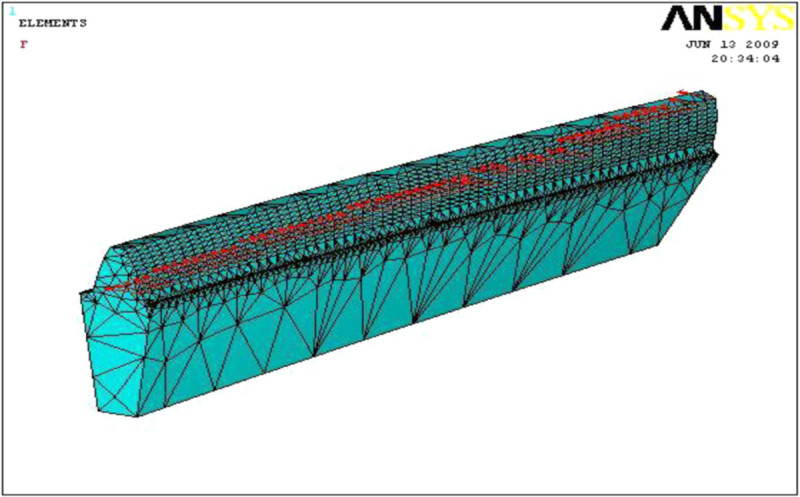
**FEA meshed model of a single tooth.**

The gear tooth is meshed in 3 dimensional (3-D) SOLID 20 nodes 186 elements with fine mesh (size 3). SOLID186 has a quadratic displacement behavior and is well suited to model irregular meshes. The material properties chosen for analysis are presented in Table 5. In order to facilitate the finite element analysis the gear tooth is considered as cantilever beam and tooth force is applied diagonally along the line of contact as shown in Figures 7 and 8. Besides, same number of elements was selected and the loading was followed for the entire three models during the Finite Element Analysis for the better results.

**Table 5:** **Material properties**

Gear material	Alloy structural steel
Density	7870 kg/m^3^
Young’s modulus	206 GPa
Poisons ratio	0.3
Yield strength	637 MPa

Further, the maximum tooth bending stress (σ) for the particular pinion speed is calculated (Table 6) using the Lewis formula (Equation 8) and are compared with the ANSYS result (Shigley 2008).

**Table 6:** **Lewis maximum bending stress values**

Speed r/min	Maximum bending stress (N/mm^2^)
509.2	432.180

8 $$\sigma =\frac{K_{y}\;x\;F_{t}}{\;b\;x\;Mn\;x\;Y\;}$$

Where, $K_{y}=\sqrt{\frac{5.56+\sqrt{V}}{\;5.56\;}},$, V = π × d × N/60,000 (m/s) and

*Y=* Lewis form factor

## Discussion and evaluation

**Figure 9:**
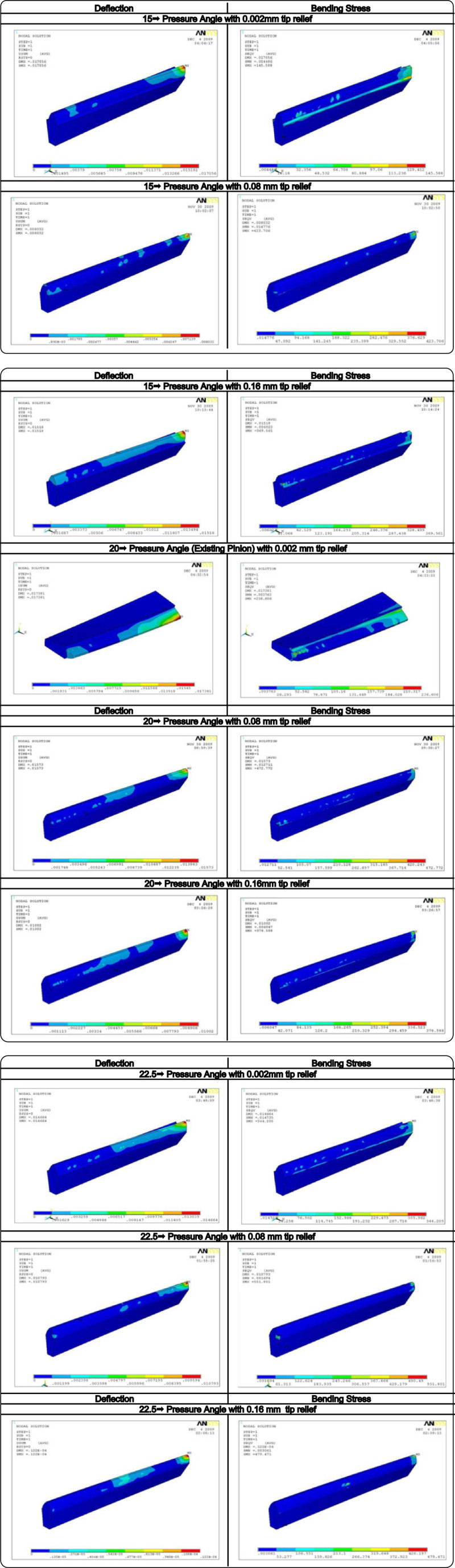
**ANSYS resultsc.**

**Figure 10:**
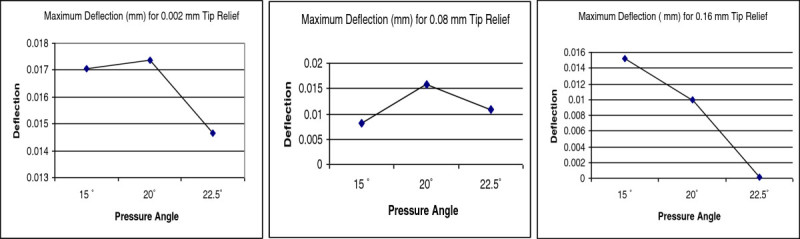
**Deflection comparison graph.**

In this paper a comparative study was carried out between three different pressure angles to select an appropriate profile to avoid frequent failure of pinion used in the gearbox of wind turbine generator. The analysis was carried out after introducing tip relief amount of 0.002 mm, 0.08 mm and 0.16 mm to the pinions in ANSYS. The induced bending stress and deflection (Figure 9) in 24 teeth pinion provided with known tip relief for different pressure angle and the calculated stiffness for the corresponding tangential force are presented in Table 7. Figure 10 shows the comparison plot between deflection and pressure angles while the pinion is subjected to load. It is obvious from Table 7 that the pinion having 20° pressure angle with 0.002 mm tip relief experience 236.606 N/mm^2^ bending stress and 0.017381 mm deflection. Similarly, pinion having 15° pressure angle with 0.002 mm tip relief have undergone approximately the same deflection (0.017056 mm) but least bending stress (145.588 N/mm^2^) among the other models; whereas the deflection is minimum (0.000122 mm) in pinion having 22.5° pressure angle with 0.16 mm tip relief but the induced bending stress (479.471 N/mm^2^) is above the Lewis maximum bending stress (432.180 N/mm^2^). It is also understood from Table 7 that only the pinion having 15° pressure angle with 0.002 mm tip relief and 20° pressure angle with 0.002 mm tip relief are experiencing lesser bending stress (145.588 N/mm^2^ and 236.606 N/mm^2^) than the Lewis maximum bending stress (432.180 N/mm^2^).

**Table 7:** **FEA results**

Pinion no of teeth	Pressure angle and contact ratio	Tip relief (mm)		Maximum deflection (mm)	Maximum bending stress (N/mm^2^)	Stiffness (N/mm)
24	20°		Ca = 0.002	0.017381	236.606	13.83×10^6^
	2.948	(i)	C_a_ =0.08	0.01573	472.772	15.28×10^6^
			∆L_a_ = 2.40			
		(ii)	C_a_ = 0.16			
			∆L_a_ = 4.80	0.01002	378.588	24.00×10^6^
	15°		Ca = 0.002	0.017056	145.588	14.10×10^6^
	3.117	(i)	C_a_ = 0.08			
			∆L_a_ = 2.40	0.008032	423.706	29.94×10^6^
		(ii)	C_a_ = 0.16			
			∆L_a_ = 4.80	0.01518	369.561	15.84×10^6^
	22.5°		Ca = 0.002	0.014664	344.205	16.42×10^6^
	2.878	(i)	C_a_ = 0.08			
			∆L_a_ = 2.40	0.010793	551.801	22.28×10^6^
		(ii)	C_a_ = 0.16			
			∆L_a_ = 4.80	0.000122	479.471	2404×10^6^

Further, it is obvious from force analysis topic that the factors influencing for gear failure such as undercut and interference problems are very well considered in this design calculation. So, it is evident from the study that the frequent pinion failure is not because of wrong selection of minimum number of teeth.

**Figure 11:**
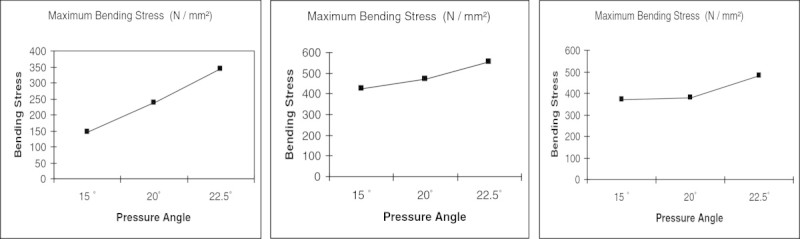
**Bending stress comparison graph.**

Further, it is observed from the plot (Figure 10) that the tooth deflection is in down trend for pinion with 0.16 mm tip relief with increase in pressure angle. However, it is different in nature for the 0.002 mm and 0.08 mm tip relief. Looking in to the induced bending stress comparison graph (Figure 11); the helical pinion having 22.5° pressure with 0.16 mm tip relief is around 479.471 N/mm^2^ which is higher than the Lewis maximum bending stress (432.180 N/mm^2^). Further, among the entire model, pinion having 22.5° pressure angle with 0.08 mm tip relief undergone maximum bending stress (551.801 N/mm^2^). The above analysis and investigation have been done without changing the operational environment (power, speed ratio and other critical design specifications).

## Conclusions

Based on the results obtained in this study, the following conclusions can be drawn,

1. It is obvious from Table 7 that only the pinions having 15° pressure angle with 0.002 mm tip relief and 20° pressure angle with 0.002 mm tip relief are experiencing lesser bending stress than Lewis maximum bending stress. Among the two models, low pressure angle helical pinion (15° pressure angle with 0.002 mm tip relief) running at slow speed(509.2 rpm) provide improved performance with lesser bending stress (145.588 N/mm^2^) over more traditional 20° pressure angle pinion (236.606 N/mm^2^). This was verified through ANSYS analysis.
2. Even though the 20° pressure angle pinion have many practical advantages such as it reduces the risk of undercut, it has greater length of contact and stronger at root, it is evident from Figure 2 that because of more sharp and weaker at the tip when compared to the modified pinion (15° pressure angle) the traditional pinion (20° pressure angle) undergone breakage of tooth only at the tip portion in all the cases.
3. The study infers that the 15° pressure angle pinion (contact ratio 3.117) is a superior choice for slow speed stage of gearbox used in the wind turbine generator. Here the author’s recommendation to avoid frequent pinion failure is that instead of using 20° pressure angle gear pair in both slow speed and high speed stage the traditional gear pair (20° pressure angle) having contact ratio 2.948 can be used only at high speed stage as the high-pressure angle gears are most efficient when operated in the high speed.

## Authors’ original submitted files for images

Below are the links to the authors’ original submitted files for images. Authors’ original file for figure 1 Authors’ original file for figure 2 Authors’ original file for figure 3 Authors’ original file for figure 4 Authors’ original file for figure 5 Authors’ original file for figure 6 Authors’ original file for figure 7 Authors’ original file for figure 8 Authors’ original file for figure 9 Authors’ original file for figure 10 Authors’ original file for figure 11

## Abbreviations

Z: Number of teeth
M_n_: Normal module
α: Normal pressure angle
β: Helix angle
d: Pitch circle diameter
d_b_: Base circle diameter
a: Centre distance
d_a_: Tip circle diameter
x: Addendum modification co-efficient
d_r_: Root circle diameter
h_a_: Addendum
h_f_: Deddendum
C_a_: Permissible tip relief amount
∆L_a_: Allowable tip relief length
F_a_: Axial force
F_r_: Radial force
F_t_: Tangential force
FEA: Finite element analysis
WTG: Wind turbine generator.
