# Supplementary figures and images for: Effects of pressure angle and tip relief on the life of speed increasing gearbox: a case study
Source: Springerplus. 2014 Dec 16;3:746. doi: 10.1186/2193-1801-3-746 (PMC4320157; doi:10.1186/2193-1801-3-746)

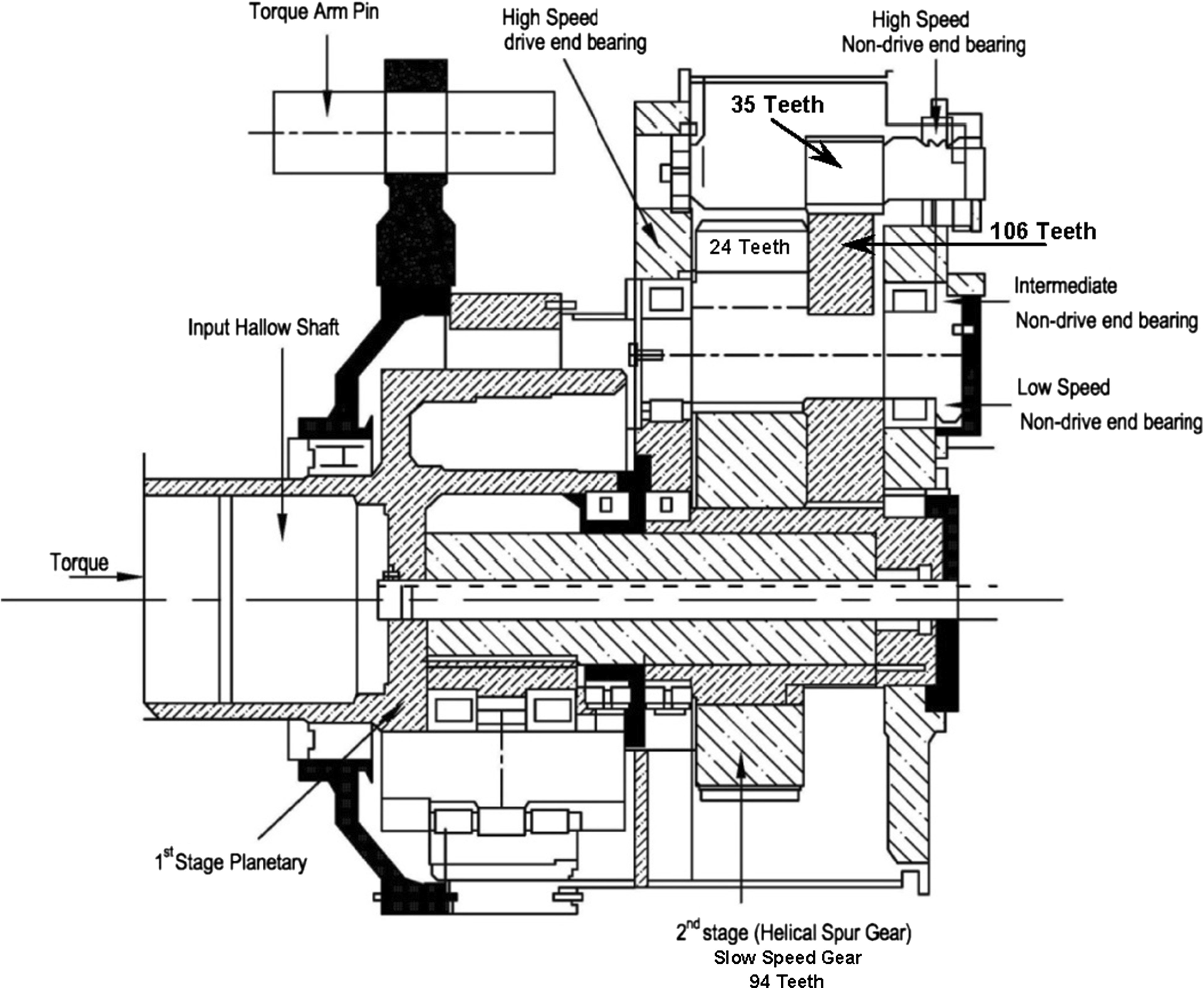

Supplement: Supplementary file 1 — Authors’ original file for figure 1 [file 40064_2014_1509_MOESM1_ESM.tiff]

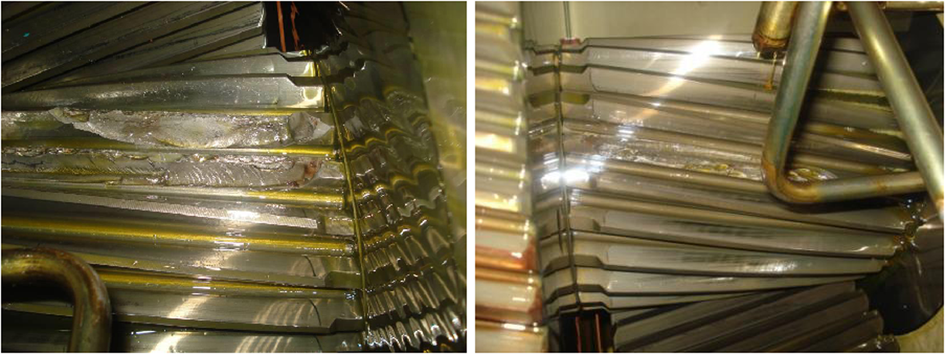

Supplement: Supplementary file 2 — Authors’ original file for figure 2 [file 40064_2014_1509_MOESM2_ESM.tiff]

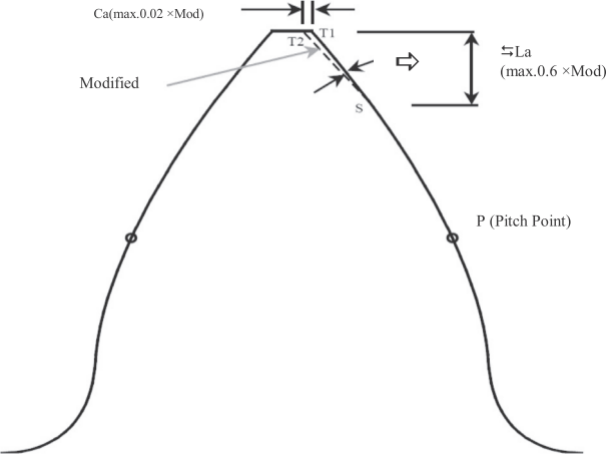

Supplement: Supplementary file 3 — Authors’ original file for figure 3 [file 40064_2014_1509_MOESM3_ESM.pdf]

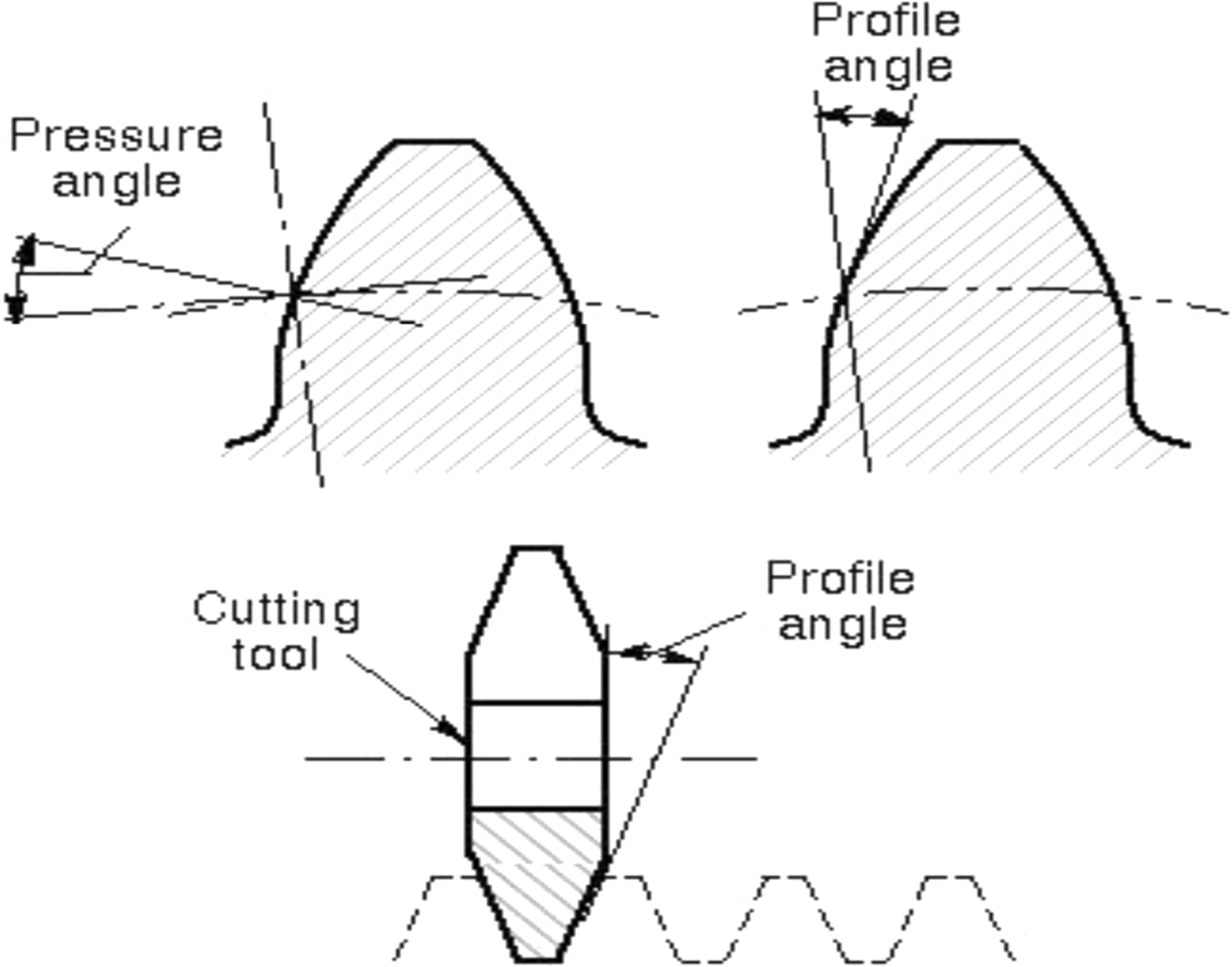

Supplement: Supplementary file 4 — Authors’ original file for figure 4 [file 40064_2014_1509_MOESM4_ESM.tiff]

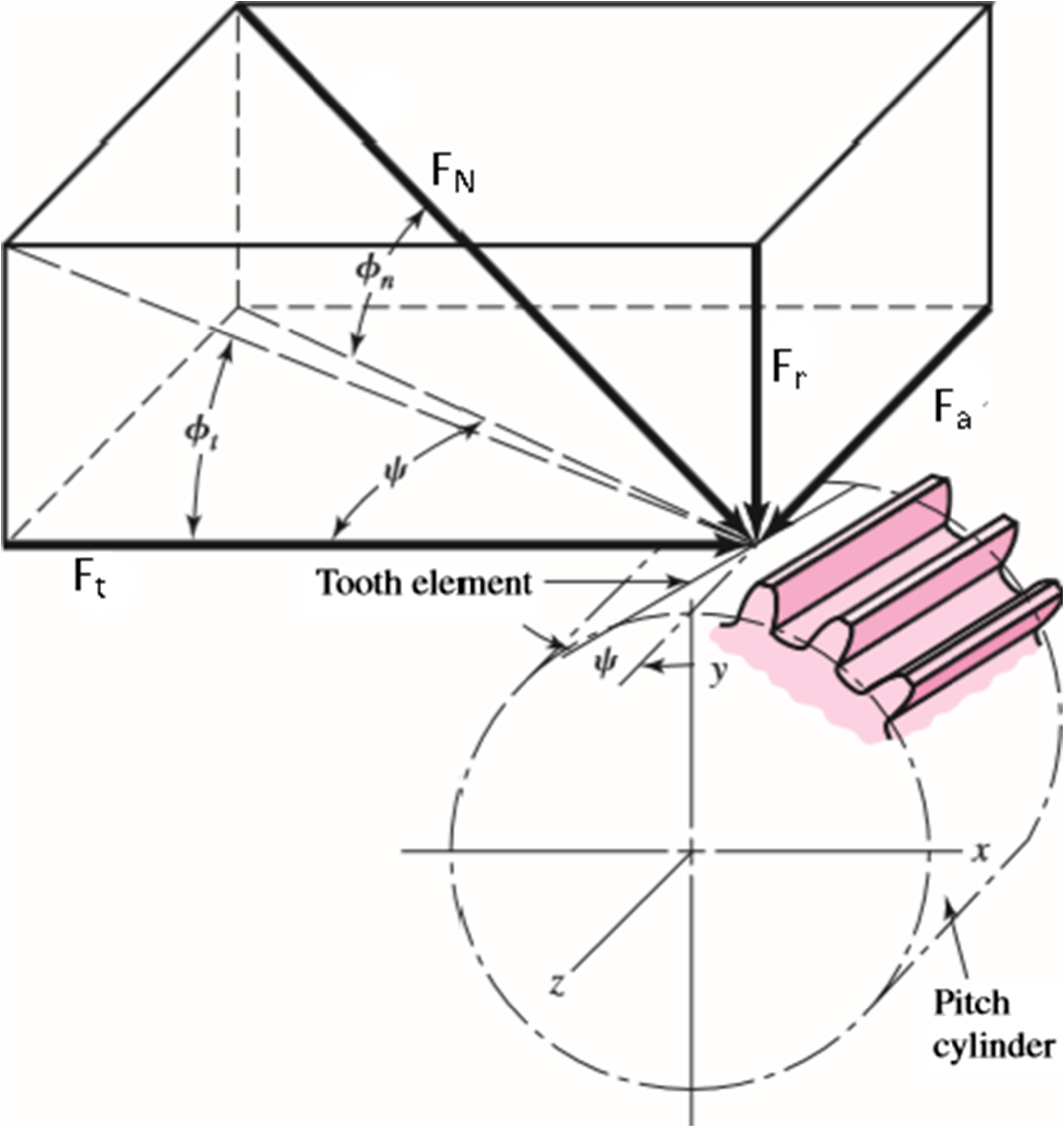

Supplement: Supplementary file 6 — Authors’ original file for figure 6 [file 40064_2014_1509_MOESM6_ESM.tiff]

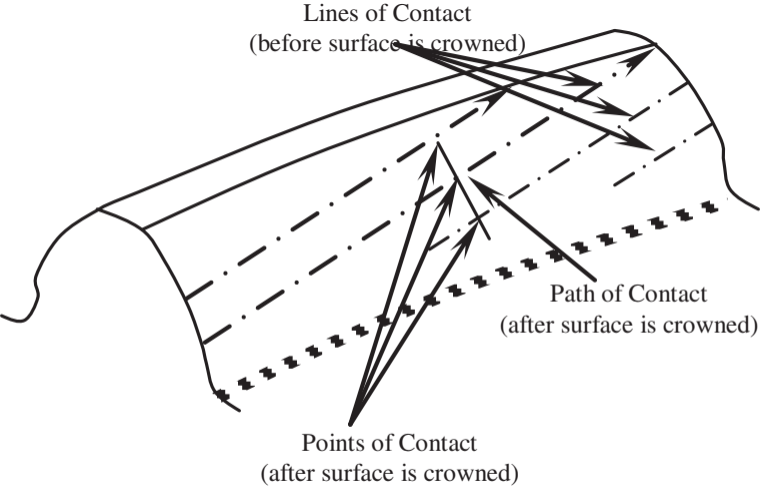

Lines of Contact (Helical Gear) ANSI/AGMA 1012-G05

Supplement: Supplementary file 7 — Authors’ original file for figure 7 [file 40064_2014_1509_MOESM7_ESM.pdf]

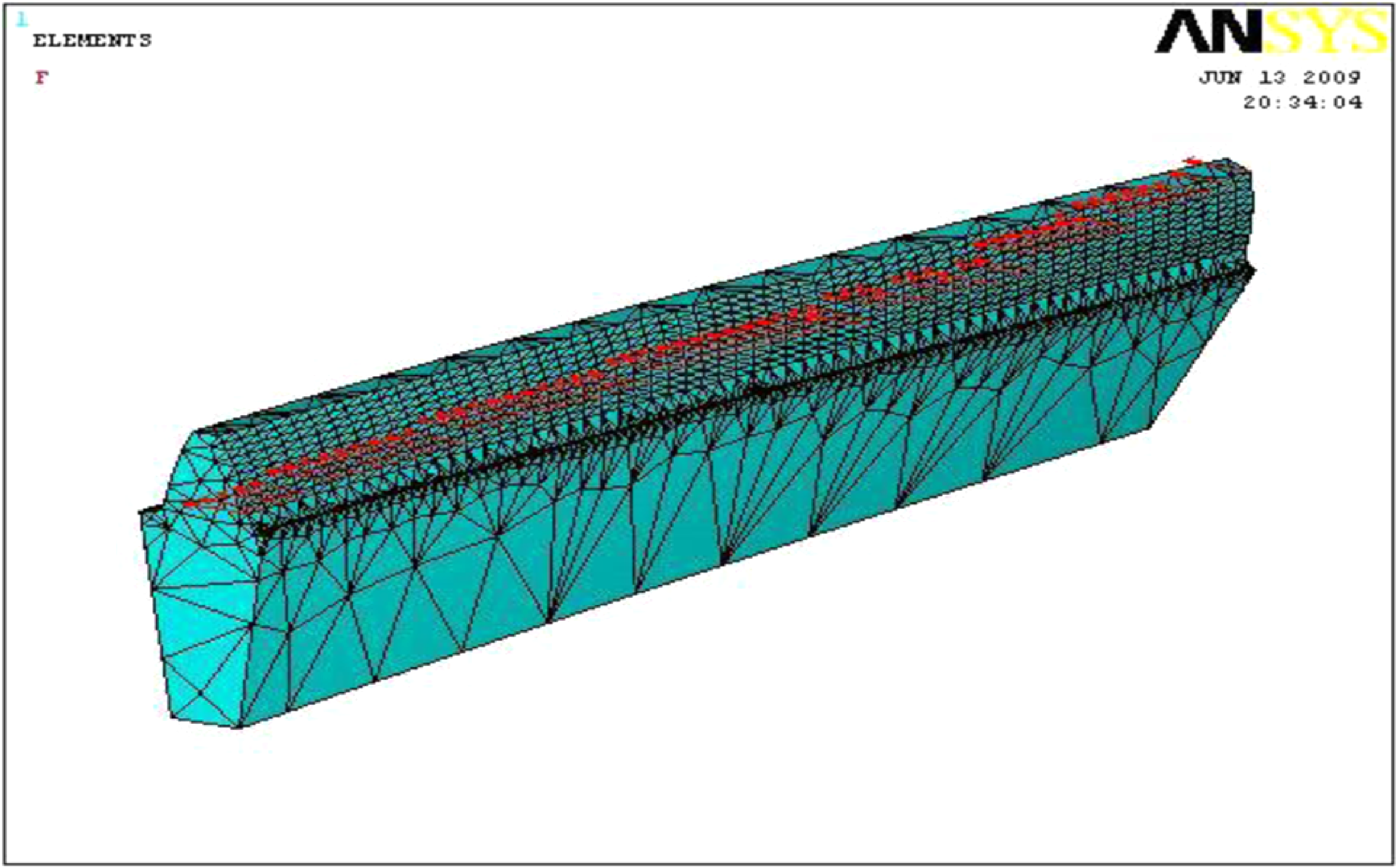

Supplement: Supplementary file 8 — Authors’ original file for figure 8 [file 40064_2014_1509_MOESM8_ESM.tif]

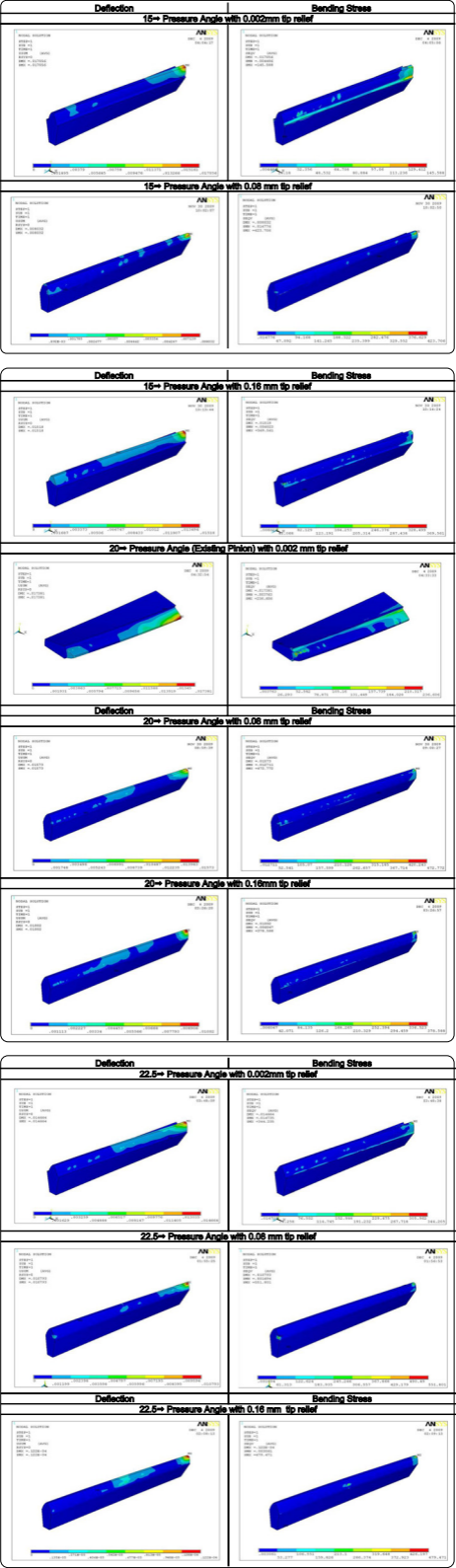

Supplement: Supplementary file 9 — Authors’ original file for figure 9 [file 40064_2014_1509_MOESM9_ESM.pdf]
