# Supplementary material for: Effects of pressure angle and tip relief on the life of speed increasing gearbox: a case study
Source: Springerplus. 2014 Dec 16;3:746. doi: 10.1186/2193-1801-3-746 (PMC4320157; doi:10.1186/2193-1801-3-746)
Supplement: Supplementary file 5 — Authors’ original file for figure 5 [file 40064_2014_1509_MOESM5_ESM.pdf]

15° Pressure Angle		
0.002 mm Tip Relief	0.08 mm Tip Relief	0.16mm Tip Relief
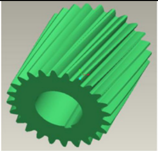	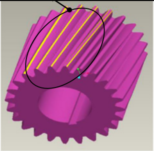	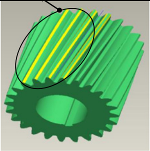
20° Pressure Angle		
0.002 mm Tip Relief	0.08 mm Tip Relief	0.16mm Tip Relief
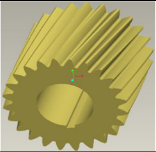	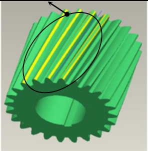	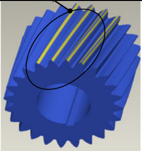
22.5° Pressure Angle		
0.002 mm Tip Relief	0.08 mm Tip Relief	0.16mm Tip Relief
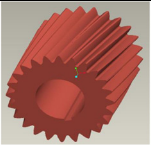	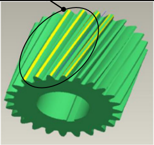	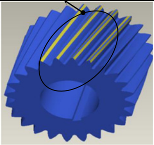
