# Supplementary material for: Effects of pressure angle and tip relief on the life of speed increasing gearbox: a case study
Source: Springerplus. 2014 Dec 16;3:746. doi: 10.1186/2193-1801-3-746 (PMC4320157; doi:10.1186/2193-1801-3-746)
Supplement: Supplementary file 10 — Authors’ original file for figure 10 [file 40064_2014_1509_MOESM10_ESM.pdf]

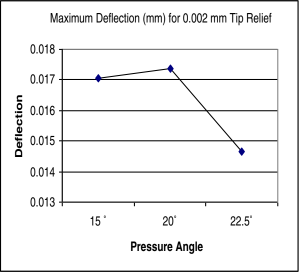

Maximum Deflection (mm) for 0.002 mm Tip Relief
0.018
0.017
0.016
0.015
0.014
0.013
Deflection
15 °
20°
22.5°
Pressure Angle

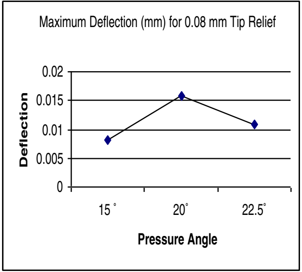

Maximum Deflection (mm) for 0.08 mm Tip Relief
0.02
0.015
0.01
0.005
0
Deflection
15 °
20°
22.5°
Pressure Angle

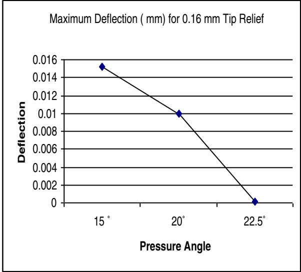

Maximum Deflection (mm) for 0.16 mm Tip Relief
0.016
0.014
0.012
0.01
0.008
0.006
0.004
0.002
0
Deflection
15 °
20°
22.5°
Pressure Angle
