# Supplementary material for: Effects of pressure angle and tip relief on the life of speed increasing gearbox: a case study
Source: Springerplus. 2014 Dec 16;3:746. doi: 10.1186/2193-1801-3-746 (PMC4320157; doi:10.1186/2193-1801-3-746)
Supplement: Supplementary file 11 — Authors’ original file for figure 11 [file 40064_2014_1509_MOESM11_ESM.pdf]

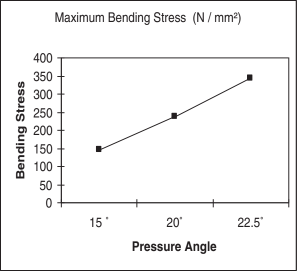
Maximum Bending Stress (N / mm²)
Bending Stress
400
350
300
250
200
150
100
50
0
15 °
20°
22.5°
Pressure Angle

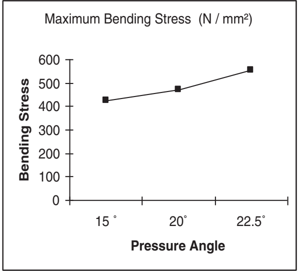
Maximum Bending Stress (N / mm²)
Bending Stress
600
500
400
300
200
100
0
15 °
20°
22.5°
Pressure Angle

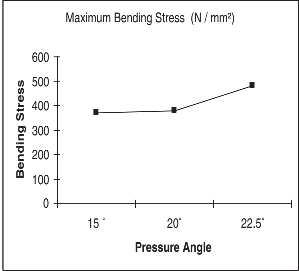
Maximum Bending Stress (N / mm²)
Bending Stress
600
500
400
300
200
100
0
15 °
20°
22.5°
Pressure Angle
